# Multiplex Immunoassay Techniques for On-Site Detection of Security Sensitive Toxins

**DOI:** 10.3390/toxins12110727

**Published:** 2020-11-20

**Authors:** Christopher Pöhlmann, Thomas Elßner

**Affiliations:** Bruker Optik GmbH, Permoserstr, 15, 04318 Leipzig, Germany; Thomas.Elssner@bruker.com

**Keywords:** security sensitive toxins, multiplex immunoassay platforms, on-site detection, optical biosensor, electrochemical biosensor, proteotoxins, low molecular weight toxins

## Abstract

Biological toxins are a heterogeneous group of high molecular as well as low molecular weight toxins produced by living organisms. Due to their physical and logistical properties, biological toxins are very attractive to terrorists for use in acts of bioterrorism. Therefore, among the group of biological toxins, several are categorized as security relevant, e.g., botulinum neurotoxins, staphylococcal enterotoxins, abrin, ricin or saxitoxin. Additionally, several security sensitive toxins also play a major role in natural food poisoning outbreaks. For a prompt response to a potential bioterrorist attack using biological toxins, first responders need reliable, easy-to-use and highly sensitive methodologies for on-site detection of the causative agent. Therefore, the aim of this review is to present on-site immunoassay platforms for multiplex detection of biological toxins. Furthermore, we introduce several commercially available detection technologies specialized for mobile or on-site identification of security sensitive toxins.

## 1. Introduction

Security sensitive toxins comprise a heterogeneous group of high and low molecular weight substances produced by living organisms and are noted for their ability to incapacitate or decimate human, animal and plant hosts. Their common occurrence, ease of dissemination as well as the difficulty in their identification due to common illness symptoms after intoxication are attributes to make them potential biological warfare agents (BWAs). Therefore, biological toxins are “chemical agents” yet of biological origin, exhibiting partly also enzymatic activity (also so-called “mid spectrum agents”) [1].

Security-sensitive toxins can be divided into two subgroups, i.e., high molecular (also known as proteotoxins) and low molecular weight toxins. Furthermore, biological toxins can be classified according to their producing organism (e.g., bacteria, plant, snake). Among the group of high molecular weight toxins, the most prominent representatives are botulinum neurotoxins (BoNTs), produced by the bacterium *Clostridium botulinum*, staphylococcal enterotoxins (SEs), produced by the bacterium *Staphylococcus aureus*, and the plant toxins ricin (from *Ricinus communis*) and abrin (from *Abrus precatorius*); whereas the most prominent representatives for the group of low molecular weight toxins are the potent neurotoxin saxitoxin, produced by marine dinoflagellates, the carcinogenic mycotoxins aflatoxins, produced by several molds, or the trichothecene mycotoxin T-2. Table 1 summarizes security sensitive toxins, showing their biological potency in comparison to exemplarily synthetic chemical agents.

Since the aim of this review is to give a technological overview of suitable multiplex on-site detection methodologies for security relevant toxins, we do not here give a detailed description of the particular toxins mentioned in Table 1. However, we refer to several reviews describing their particular characteristics and the use of biological toxins as potential biothreat agents [6,7,8].

Biological toxins have been exploited throughout history as BWAs as well as bioterrorism agents to cause physical damage as well as to create fear and panic in the human population [9,10]. There is a broad spectrum of bioterrorism, ranging from hoaxes and deliberate release of non-mass casualty agents by individuals or small groups to state-sponsored terrorism employing classical BWAs which can cause large scale outbreaks and mass casualties [11]. The terror caused by the use of BWAs led finally to the Biological and Toxins Weapon Convention (BTWC) in 1972, which currently involves 183 states parties committing to the prohibition of the development, production and stockpiling of biological and toxin weapons. The BTWC seems to be effective in controlling the proliferation of BWAs at state level; however, it is ineffective in avoiding terroristic attacks by individuals and small groups using BWAs. In the past, the proteotoxin ricin has been used in criminal and bioterrorism attacks, most notably in the assassination of Bulgarian dissident Georgi Markov in 1978 and mail letter attacks in the United States in 2003 and 2013 [6]. Recently, in 2018, a foiled terror attack in Cologne, Germany, when plant toxin ricin was prepared, demonstrated again the potential use of biological toxins for intentional release [12]. Thus, there is an increased demand for overall preparedness to address the challenges connected to the rapid and reliable identification as well as the diagnosis and the treatment of intoxications with security relevant toxins [13]. Successful implementation of anti-bioterrorism measures depends on the rapid and on-site simultaneous monitoring and identification of an as broad as possible panel of biothreat agents as part of a pre-warning for timely initiation of appropriate organizational as well as medical countermeasures in the case of an attack with biothreat agents [2]. Therefore, it is of utmost importance that research is conducted with the aim of developing reliable detection technologies to initiate timely effective medical countermeasures. One issue with biological attacks is determining whether an attack has occurred. Initial symptoms after intoxication with biological toxins are difficult to distinguish from a natural outbreak with more benign biological agents. Therefore, a rapid identification of potential BWAs is essential.

## 2. Antibodies and Immunosensors

In contrast to infectious disease detection relying mostly on molecular techniques (nucleic acid testing (NAT)) with several commercially available NAT platforms on the market [14], there is a substantial divergence in the immunoassay platform landscape. In contrast to multiplex NAT platforms, there are significantly fewer multiplex immunoassay platforms available on the market. However, in the case of security relevant toxins, the detection of the gene encoding for the particular toxin or the identification of the producing organism are often not sufficient. The identification of the complete or parts of the gene encoding for the toxin demonstrates the capability to produce the toxin, but it is not evident that the toxin is expressed by the organism [15]. Furthermore, biological toxins can also be present in the absence of the genetic material. Particularly, the scenario of intentional release of security sensitive toxins by a terrorist entails the risk that a nucleic acid free protein solution will be released. Additionally, in clinical matrices such as serum, there is a potential risk that only the toxin is detectable and not the producing bacterium [16]. Because of the mentioned limitations of molecular biology-based detection techniques for toxin identification, and at the same time the inherent high specificity and sensitivity of immunological-based identification techniques, we will focus in this review on on-site multiplex immunoassay platforms and will not consider nucleic acid-based assays. Additionally, due to the focus on on-site detection techniques, we will not include any sophisticated laboratory-based platforms, such as matrix-assisted laser desorption/ionization time-of-flight mass spectrometry (MALDI-TOF MS) or LC-MS/MS methods. Recently, these techniques delivered promising results in laboratory environments [17,18,19,20,21]. Particularly, these methods are highly specific and can therefore differentiate between closely-related toxins of a toxin group. Furthermore, these methods can provide quantitative results. However, due to instrument size and weight, complexity of sample preparation (mostly done by immunoextraction steps), interpretation of results and the necessity for trained users, they are up to now not suited for on-site detection purposes.

The critical biochemical reagent of every immunoassay determining analytical performance is the used antibody. The presence of foreign antigens provokes an immune response of vertebrate immune systems, generating various chemical signaling molecules as well as proteins to combat the invading antigens and, therefore, weaken the infection. Antibodies, also known as immunoglobulins (Igs), secreted by activated B cells, represent a main part of the adaptive immune system capable of binding foreign antigens and sequestering them until they can be removed from the host through phagocytosis or other mechanisms. One extraordinary characteristic of antibodies is their incredibly high degree of specificity exhibiting binding constants in the nanomolar range or better [22]. An alternative to conventional antibodies, heavy chain antibodies (hcAbs) consisting only of two heavy chains found in camelids, have raised considerable attention in the field of on-site biosensing approaches due to their unique properties, particularly their small size and extraordinary stability [23]. In the past, fragments of hcAbs, so-called single-domain antibodies (sdAbs) or nanobodies (only variable region of single heavy chain), have already been employed for proteotoxin detection, e.g., for the specific determination of cholera toxin [24] or ricin [25]. However, due to the necessity of more sophisticated manufacturing processes for these fragment antibodies and accompanying potential higher manufacturing costs, fragment antibodies or nanobodies did until now not find their way heavily into applications in multiplex on-site detection assays.

**Figure 1:**
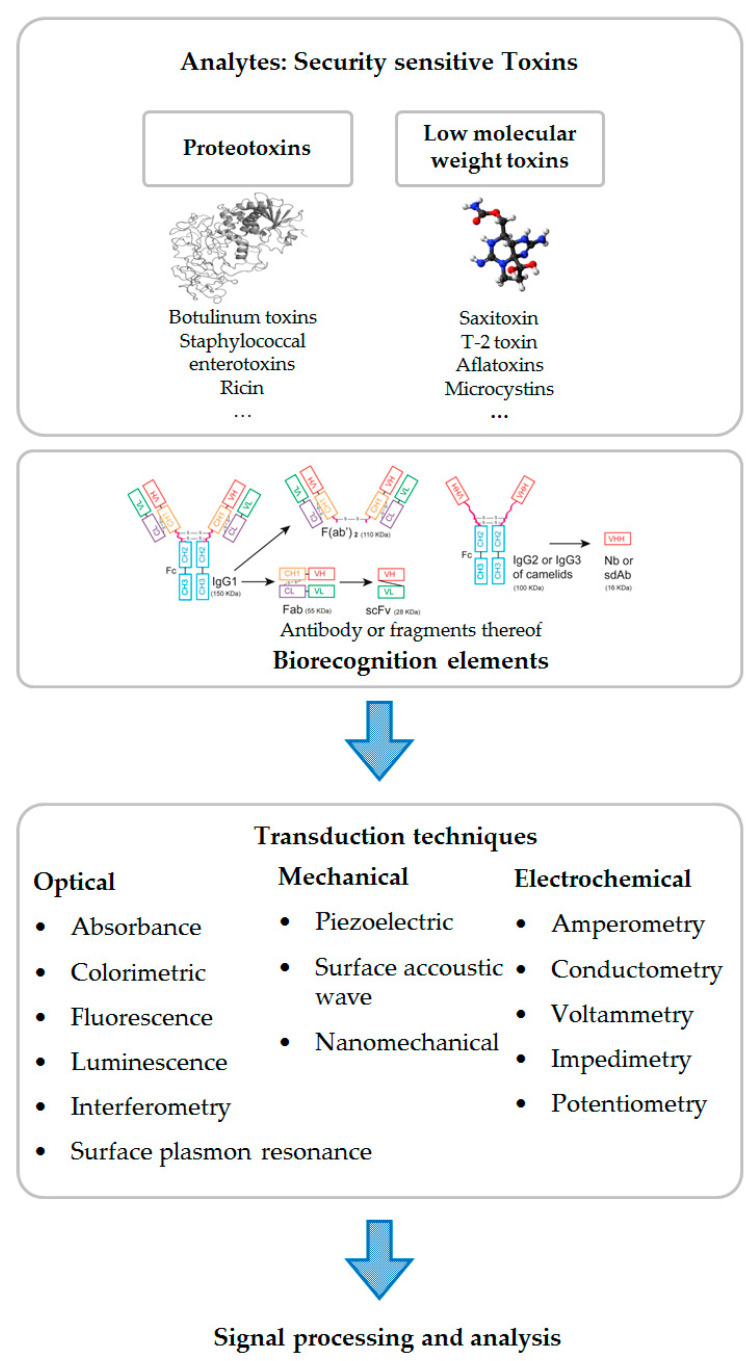
General overview of immunosensor components for detection of security sensitive toxins as analyte (3D structure model of ricin (pdb: 2AAI [30]) as well as a ball-and-stick model of saxitoxin are depicted). Because this review is focused on immunosensors, antibodies (or fragments of antibodies) (VL: variable light chain; VH: variable heavy chain; CL: constant light chain; CH: constant heavy chain; Fab: antigen binding fragment; scFv: single-chain variable fragment; VHH: variable domain of heavy chain antibody; Nb: nanobody; sdAb: single domain antibody) are depicted as biorecognition elements only (Adapted from [31]. MDPI (2014)). For the sake of completeness, it should be noted that alternative biorecognition elements, such as aptamers, natural receptor proteins, carbohydrates or cell-based receptors, could also be employed for toxin detection. Several examples of possible transduction mechanisms are noted as well as signal processing.

An immunosensor is a biosensor applying antibodies as biorecognition elements, and thus exploiting antibody–antigen interaction for specific detection of a particular analyte in complex matrices (Figure 1) [26]. The IUPAC (International Union of Pure and Applied Chemistry) definition of a biosensor is “a device that uses specific biochemical reactions mediated by isolated enzymes, immunosystems, tissues, organelles or whole cells to detect chemical compounds usually by electrical, thermal or optical signals” [27]. The majority of immunoassay methods are heterogeneous immunoassays, i.e., one reaction component (antigen or antibody) is immobilized on a solid support (e.g., polystyrene of microtiter plate, magnetic beads, gold electrodes of a biosensor). Homogeneous immunoassays manage to discriminate bound from unbound labels without a separation step simplifying assay procedure, i.e., only mixing of a sample and immunochemical reagents is required followed by detection. The binding of the analyte to the immunochemical reagent results instantly in a physically detectable signal [28,29]. The advantages of homogeneous immunoassays, such as very fast incubation times, minimal operator intervention, obviating the requirement for wash steps and hence facilitating maximum speed, are accompanied with the disadvantage of susceptibility towards interferences by complex sample matrices. Furthermore, competitive and non-competitive immunoassay formats can be differentiated.

In direct immunoassay formats, the antigen is immobilized on the solid support allowing for the determination of the amount of binding antibodies. Similar to direct immunoassays, in indirect immunoassays the antigen is immobilized on the solid support. However, indirect format requires the presence of a secondary antibody which recognizes the first antibody bound to the antigen [32]. In sandwich immunoassays, an antibody is immobilized on the solid support. In a first step, the analyte binds to the immobilized antibody; in a subsequent second step, a detection antibody binds to the bound antigen and allows for the generation of a detectable signal. This assay is mostly applied for detection of macromolecules such as proteotoxins having at least two antigenic determinants for separate binding of capture and detection antibody (non-competitive immunoassay format). In contrast, competitive immunoassay format is usually used for the detection of low molecular weight toxins, such as saxitoxin or microcystins, exhibiting only one epitope for antibody binding. In competitive immunoassay formats, the analyte competes with an antibody in solution for binding to the antigen immobilized on a solid support.

The second step of an immunoassay is the signal generation (signal transduction) after primary antibody–antigen interaction. Antibodies or antigens have to be labeled either directly or indirectly to ensure sensitive detection of the antibody–antigen interaction. Indirect labeling approaches apply either functionalized secondary antibodies or the biotin–avidin system requiring an additional noncovalent binding step applying streptavidin-conjugated reporter enzymes. Typical reporter enzymes include horseradish peroxidase (HRP), alkaline phosphatase (AP) or ß-galactosidase (ß-Gal). Furthermore, the application of thermostable reporter enzymes such as Esterase 2 from *Alicyclobacillus acidocaldarius* results in increased stability, improved sensitivity or a more straightforward assay workflow, as demonstrated for nucleic acid-based electrochemical sensors [33,34]. In combination with the usage of the above described sdAbs or nanobodies as immunochemical reagents, the application of thermostable reporter enzymes promises in the future potential particularly for applications in on-site immunoassays because of required robustness towards environmental influences (e.g., operation and storage temperature, shelf life of the assay kits; see also Table 2). Further signal enhancement can be achieved using, for example, polymers carrying several units of HRP [35], dendrimeric structures carrying several units of a particular reporter enzyme [36] or click chemistry-mediated assembly of multiple HRPs together with detection antibodies [37].

Another promising signal amplification technique for immunoassays is immuno-PCR (iPCR), applying DNA-labeled detection antibodies [38,39]. After binding of the detection antibody to the target molecule, the oligonucleotide tag is amplified by PCR achieving sensitivities in the low pg/mL or even fg/mL range for proteotoxins such as ricin [40], SEs [41], BoNT/A [42] and /B [43] as well as low molecular weight toxins like aflatoxins [44] or zearalenone [45]. Until now, the application of iPCR techniques is mostly limited to single-plex or 2-plex reactions, assay times are mostly significant longer than 60 min and demonstration of robustness towards a wide range of sample matrices is limited. Therefore, iPCR is a promising signal amplifications strategy; however, its wider applicability—particular its robustness for application in on-site detection—will have to be shown in the future.

Recently, a CRISPR/Cas13a-based signal amplification strategy was described for the sandwich immunoassay-based detection of an inflammatory factor (human interleukin-6) and a tumor marker (human vascular endothelial growth factor) achieving a limit of detection (LOD) in the low fM-range [46]. However, the strong signal amplification effect (approx. factor 100 compared to classical ELISA) was accompanied with a more complex assay workflow (more reagent additions, more washing steps) as well as an elongated assay time of approx. 2 h compared to the classical ELISA. In summary, from a general point of view, signal amplification strategies have been up to now mostly applied to single-plex immunoassays for detection of model antigens or in nucleic acid-based biosensors but have not found their way in the on-site multiplex detection of security sensitive toxins.

Beside enzymatic labels, fluorophores are applied for fluorescence detection. Innovative fluorescence labels such as quantum dots (QDs) [47] or lanthanide-doped nanoparticles [48] have contributed already significantly to the field of multiplex detection and their influence will certainly increase in the future. Goldman et al. [49] developed a 4-plex immunoassay for detection of cholera toxin, ricin, shiga-like toxin1 and staphylococcal enterotoxin B (SEB). Capture antibodies for the four different toxins were immobilized on the surface of a microtiter plate in a single well, whereas detection antibodies were conjugated to four different colored QDs. The four different QD colors exhibit emission maxima separated by at least 20 nm allowing for sufficient spectral resolution. In contrast to indirect protocols, direct labeling protocols use conjugation of common reporter enzymes or fluorophores to primary antibodies. Direct protocols suffer often from decreased antibody—reporter enzyme conjugate affinity and stability due to side effects of cross-linking chemistry [31]. Use of recombinant antibody technology to generate antibody—reporter enzyme fusion proteins genetically allows improved production of these conjugates without the limitations of affecting the antigen binding site by the cross-linking reagent [50]. Compared to noncovalent binding involved in indirect protocols, direct labeling protocols offer several advantages, such as more straightforward assay workflow due to the avoidance of a further binding step, more rapid detection of the analyte and often improved sensitivity. However, directly labeled immunochemicals or recombinantly engineered antibody fusion proteins are not yet widely applied, mainly because of more sophisticated production protocols and accompanying increased manufacturing costs.

In the final step, the readout signal is generated. Common transduction techniques for immunosensors are optical (including fluorescence and luminescence as well as label-free techniques, such as surface plasmon resonance), mechanical (e.g., surface-stress mechanical biosensors, quartz crystal microbalance, whispering-gallery microgravity), mass-sensitive or electrochemical (e.g., potentiometric, amperometric, impedimetric biosensors) readout techniques [51].

## 3. Criteria for On-Site Application of Multiplex Immunoassay Techniques

The scope of this review is to give an updated comprehensive overview about technological developments happening in the field of on-site and multiplex toxins detection. This will include commercial available on-site techniques as well as commercial immunoassay platforms or prototypes in development which show in our opinion technological feasibility to be re-designed with some considerable effort to be applied for on-site detection. The development of multiplex immunosensor platforms capable of being applied in the field requires special characteristics which should be discussed in this chapter in more detail.

In 2006, Peeling et al. [52] set the criteria for applicability of clinical diagnostics tests to resource-limited settings using the term ASSURED: Affordable, Sensitive, Specific, User-friendly, Rapid and robust, Equipment-free and Deliverable. In our opinion, the majority of these criteria are also transferable to on-site useable multiplex immunoassay-based methodologies for detection of security sensitive toxins. Often overlooked is the fact that, beside analytical parameters, operational characteristics of a detection technology are crucial for the final success of this technique in real life application. Due to the fact that the devices aimed to be used by first responders having a variety of other tasks beside identification of BWAs in their daily work life, these platforms have to be as easy-to-use and robust as possible. Furthermore, the stability of the immunoreagents towards extreme temperatures as well as humidity, the low weight of the detection system (portability), the capability to analyze a sample for several agents (multiplexing) and, optimally, the possibility to analyze several samples in parallel (throughput) all have to be added to the ASSURED term. Particularly, the capability of multiplex testing is of utmost importance, because multiplexing reduces costs and enhances efficiency of testing [53]. Multiplexing is requested by first responders coming to a scene when the type of agent released is completely unknown as well as when the sample is limited.

An overview of several key characteristics of portable platforms for biological toxin detection is depicted in Table 2. The selection of the mentioned key parameters as well as the classification in the categories “Minimal requirement” and “Optimal requirement” are clearly a consensus of the personal opinion of the authors. However, for the selection and classification of the parameters, we oriented ourselves on previously published reports for immunoassay platforms designed for clinical diagnostic applications in resource-limited settings [54,55], which are in our opinion for many features adaptable to platforms for on-site detection of security sensitive toxins.

## 4. Portable Immunoassay Techniques with Multiplexing Capability

We recognize the many other excellent publications that precede this one and, in fact, were supported strongly by them in many cases. Particularly, the reviews by Gooding [56], Mirski et al. [57], Moran et al. [58], Dorner et al. [59], and Walper et al. [32] provide an overview of various technologies used for detection of biothreat agents in environmental and food samples as well as for clinical diagnostics. In the following sections, different immunoassay-based multiplex techniques for on-site detection of security sensitive toxins are presented.

### 4.1. Lateral Flow Assays

There is an increasing focus of research on innovation predominantly in lateral flow (immunochromatographic) immunoassays (LFIAs) and much less in bench-top or portable systems. LFIAs require only the addition of the sample initiating a series of reactions which result in a readable signal. In the most common sandwich format, the sample solubilizes and mobilizes a labeled antibody. The sample together with the antibody move along a chromatography membrane and bind to their particular capture site, where the label forms a visible signal. LFIAs exhibit several advantages, such as simplicity, low cost and enabling flexibility for analyte panels. The first LFIA was described and patented in the late 1970s [60,61]. In the beginning, LFIAs were developed for detection of one analyte (e.g., for determination of human hormone chorionic gonadotropin levels in urine allowing pregnancy testing [62]). Recently, several approaches for multiplex testing with LFIAs have been developed. These include several spatially separated detection sites in a single strip, aligning several strips in one array format, the application of several different signal reporters or broad-specific antibodies binding to various compounds of a class [61]. Several companies launched multiplex LFIAs for detection of security sensitive toxins (Table 3). The analysis time (run time) for all commercial LFIAs is approx. 15 min. Portable optical readers are usually offered as an accessory, often justifying additional costs and operational complexity by providing quantitative data and compensating variation in human vision. Most of these detect BoNTs (serotypes A & B), ricin and SEB. The Biothreat Alert^®^ LFIA from Tetracore, Inc. (Rockville, MD, USA) exhibits additionally the feature for detection of the important plant toxin abrin. All mentioned LFIAs are based on colloidal gold as visible dyes. The RAMP^®^ system (Response Biomedical, Vancouver, BC, Canada) uses fluorescence detection, rather than visible dyes, so a fluorescence optical reader is required. However, RAMP^®^ Test Kits have only been offered in single-plex format up to now. Most LFIAs rely on colloidal gold-labeled mAb and mAbs for capturing. miPROTECT Ricin LFIAs (miprolab, Göttingen, Germany) use a glycoprotein for capturing ricin and gold nanoparticle-labeled mAbs for single-plex detection of ricin. The determined LOD was around 20 ng/mL [63]. In general, manufacturer-reported LODs or LODs achieved in validation studies are in the range of low to mid ng/mL for multiplex LFIAs [64,65]. Validation studies for multiplex LFIAs were only done to a very limited extent so far. For example, of four compared multiplex LFIAs (IMASS^TM^, RAID^TM^ 5, RAID^TM^ 8, ProStrips^TM^) the IMASS^TM^ produced false positive results for kaolin suspicious powder samples. The three other multiplex LFIAs produced no false positives with all tested suspicious powder samples (22 powder samples, triplicate measurements, *n* = 66). Furthermore, an inclusivity testing was performed using three ricin crude extracts. All of the tested LFIAs were able to detect the crude ricin preparations (6 replicate measurements). Only RAID^TM^ 8 showed one false negative for a crushed seed mash sample [65].

Particularly, single-plex LFIAs can exhibit very competitive LODs, as for example for the KDTB Gold LFIA (NBC-Sys, Saint-Chamond, France), where an LOD of 1 ng/mL for ricin applying a run time of 15 min was reported [63].

From Table 3, it is obvious that commercially available multiplex LFIAs are designed for detection of proteotoxins only. Commercially available LFIAs relying on a competitive immunoassay format for low molecular weight toxins detection are designed only for single-plex detection achieving LODs in the low to mid ng/mL range [66,67]. There is only one commercial LFIA (Scotia Rapid Test for Paralytic Shellfish Poisoning (PSP), Scotia Rapid Testing Limited, Chester Basin, Nova Scotia, Canada) for detection of saxitoxin, which was evaluated for use in biosecurity application [68], whereas the majority of these LFIAs were validated for particular application in food analysis. However, there is a great effort in the research community to establish multiplex LFIAs for determination of low molecular weight toxins. Exemplarily, a multiplex LFIA for simultaneous determination of mycotoxins aflatoxin B1, zearalenone and T-2 toxin in maize- and cereal-based animal feeds with a run time of 20 min was established [69]. The chosen immunoassay format was again a competitive immunoassay. Multiplexing was achieved by using differently colored nanoparticles. A decrease of color intensity in case of positive detection is observed by the naked eye. LODs using this multi-color LFIA were determined as 0.5 ng/mL, 2 ng/mL and 30 ng/mL for aflatoxin B1, zearalenone or T-2 toxin, respectively [69].

In the past years, research effort was undertaken to develop strategies for enhancing the sensitivity of LFIAs. One approach is to apply immunoliposomes carrying a large amount of fluorescent reporter molecules as label for the detection of security sensitive toxins. Khreich et al. [70] developed a LFIA for detection of SEB relying on sulforhodamine B encapsulated in immunoliposomes achieving an LOD of 20 pg/mL using a fluorescence readout. However, this signal amplification strategy results in a more complex assay procedure, i.e., a prolonged assay time of 30 min as well as the need for a fluorescence readout device. Additionally, mostly these kind of signal amplification strategies were shown—as for this example—for single-plex assays only. General applicability and robustness for multiplex detection has still to be demonstrated in the future.

There are tremendous developments in the field of rapid testing systems at the moment not directly applied to the detection of security sensitive toxins but to the field of clinical biomarkers or detection of causing agents of infectious diseases. It is far behind the scope of this review to summarize all these trends, but for example recently an enhanced centrifugation-assisted lateral flow immunoassay (ECLFIA) was designed to rapidly detect protein biomarkers in whole blood. The nitrocellulose membrane of the LFIA was inserted into a centrifugal disc allowing fully automated operations, including sample preparation, active lateral flow actuation, washing, and signal amplification. In contrast to a conventional LFIA this kind of controlled fluidics could hardly be performed. The sample-to-answer time for detecting human prostate specific antigen in a drop of blood (20 μL) is 15 min exhibiting a LOD of 0.028 ng/mL (21.4-fold improvement compared to that of conventional LFIA) demonstrating impressively the potential for sensitivity improvement by technological advancements [71]. The application of innovative reporter agents, e.g., nanozymes [72], up-converting phosphor technology-based lateral flow assay [73], antibody-gated indicator delivery systems [74], surface-enhanced Raman scattering (SERS) based LFIAs [75] as well as the combination with smartphone based readout [76,77] are other trends for LFIAs [78]. Tang et al. [79] applied Eu/Tb (III) nanospheres with enhanced fluorescence as labels for anti-idiotypic nanobodies and monoclonal antibodies. Application of anti-idiotypic antibodies showed potential to replace commonly used hapten-protein conjugates in competitive immunoassay format. These anti-idiotypic antibodies compete with the original hapten for binding sites of the anti-hapten antibody [80]. Anti-idiotypic antibodies of the β-type are raised against the paratope (antigen-binding site) of the primary anti-hapten antibody such displaying an “internal image” of the hapten [81]. Based on this detection approach the authors developed a time-resolved fluorescence immunochromatographic assay for duplex detection of aflatoxin B1 and zearalenone in maize and maize-based products. Applying the anti-idiotypic nanobodies as detection agents very competitive LODs of 0.05 and 0.07 ng/mL for aflatoxin B1 or zearalenone, respectively, in buffer solution were determined demonstrating the potential of the use of novel nanotechnology based labels as well as the use of stabilized and small antibody fragments [79]. The synthesis and application of magnetic quantum dot nanoparticles as signaling agents for LFIAs offers the possibility to capture and enrich target toxins from sample solutions in a first step and subsequently serve as advanced fluorescent labels for the quantitative determination of targets on the LFIA strip. The multiplex LFIA applying these magnetic quantum dot nanoparticles for detection of BoNT/A and SEB achieved LODs of 2.52 and 2.86 pg/mL, respectively, with a total analysis time of 30 min [72]. In case of integration of an automated magnetic separation procedure after sample addition in LFIA protocol this approach can have potential for on-site detection of security sensitive toxins by rudimentary trained users.

Furthermore, there was a lot of effort in research community to develop LFIAs for further security sensitive toxins. Féraudet-Tarisse et al. [82] developed a LFIA for detection of epsilon toxin from *Clostridium perfringens* (classified as category B agent according to CDC).Within a 20 min assay time, an LOD of 0.1 ng/mL clostridial epsilon toxin in buffer with visual readout was achieved. Furthermore, sensitive detection of clostridial epsilon toxin in milk and tap water was exemplarily demonstrated.

However, the use of LFIAs is often limited in applications requiring very high sensitivity representing drivers for innovation in immunoassay platforms. Furthermore, immunoassay platforms have advantages in regard to flow control, flexibility of the assay workflow as well as data management compared to LFIAs.

### 4.2. Microarray Technology

Protein microarray technology relies on a miniaturized version of the traditional ELISA whereby multiple antibodies are immobilized onto a solid surface, allowing for the simultaneous analysis of a magnitude of antigens within a single experiment. Table 4 summarizes commercially available platforms useable for mobile or on-site multiplex detection of security sensitive toxins.

#### 4.2.1. Label-Free Technologies

Among the label-free methods, the mostly utilized technique is surface plasmon resonance (SPR), representing an optical transduction technique. In SPR, a plasmon wave is measured over a metal surface, and works by applying a light through the biological sample, causing a change in the refractive index, which is used to monitor the binding reaction [22]. Until now, the majority of the described applications of SPR for security sensitive toxin detection are still restricted to laboratory settings relying on the popular commercial Biacore platform (formerly Biocore, then acquired by GE Healthcare Life Sciences, now Cytiva, Marlborough, MA, USA) [96]. However, there are some promising reports for portable and rapid SPR platforms for the screening of toxins useable in the field. Mycotoxins have been extensively analyzed in agricultural products. Multiplexed detection of six mycotoxins by SPR was demonstrated by Joshi et al. [97]. Applying a competitive immunoassay format, the authors developed a prototype portable imaging SPR (iSPR) setup with nanostructured gold as sensor surface. The mycotoxins deoxynivalenol, zearalenone (ZEN), T-2 toxin, ochratoxin A, fumonisin B1 and aflatoxin B1 were immobilized on the chip surface via amine/ovalbumine conjugates. Compared to a benchtop SPR setup, the sensitivity of the iSPR system decreases significantly; however, the iSPR approach omits the need for any prism in the SPR setup, thus allowing one to create portable devices [97]. Continuous research on label-free SPR-based detection of low molecular weight toxins was performed for the detection of marine biotoxins. Therefore, a multitoxin SPR-based immunosensor prototype capable of detecting up to 16 analytes simultaneously was applied for the detection of toxin families of paralytic shellfish toxins, diarrheic shellfish toxins as well as amnesic shellfish toxins [98,99]. This technique is compatible with simple extraction protocols for marine biotoxins and is sensitive enough to detect these toxins below the regulated limit in shellfish samples as well as in algae and seawater. The high degree of automation, the potential to create portable devices and the ease-of-use make SPR-based immunosensors a potential tool for toxin detection [100,101]. Feltis et al. [102] developed the first hand-held and field-deployable SPR biosensor for detection of security sensitive proteotoxins. Application of this prototype for ricin detection using a sandwich immunoassay format resulted in an LOD of 200 ng/mL within 10 min analysis time. This approach seems very promising to be expanded for other security sensitive toxins; however, sensitivity is still significantly decreased compared to a benchtop SPR device (LOD of 0.5 ng/mL for ricin [103]). Furthermore, the multiplex capability of the hand-held system has still to be demonstrated.

Beside SPR as optical label-free technology, cantilever devices are also applied for the multiplexed detection of security sensitive toxins. Microcantilever devices representing a mechanical transduction technique respond to surface stress changes produced by a biorecognition reaction on the sensor surface [104]. Riccardi et al. [105] developed microcantilever resonator arrays for detection of aflatoxins as well as ochratoxin A with LODs in the low ng/mL range. Cantilever or piezoelectric sensors for proteotoxin detection are also described; however, mostly as proof-of-concept studies demonstrating only the detection of one exemplary toxin, e.g., SEB [106].

Despite the many inherent advantages of label-free techniques, these still often suffer from limited sensitivity as well as enhanced matrix interference with the sensors surface compared to enzyme amplified optical or electrochemical sensors. Therefore, the majority of multiplex immunosensors, particularly for proteotoxin detection requiring extraordinary sensitivity, are based on labeled techniques.

#### 4.2.2. Optical Immunosensors

Recently, cost-effective mass fabrication feasibility of optical devices, such as complementary metal-oxide-semiconductor (CMOS) cameras for mobile phones, has revolutionized optical sensing. In the past, the design of portable devices was ascribed only to electrochemical devices; however, this changed with the developments in CMOS camera-based detectors as well as lighting and diode-based light sources [107]. In general, one of the advantages of optical biosensors compared to electrochemical sensing techniques is the fact that they are more compatible with continuous monitoring: i.e., optical sensors seem to be appropriate for the design of wearable sensors. In contrast, electrodes exposed to complex samples tend to foul over time, resulting in decreased sensitivity for a particular target analyte [108].

##### Colorimetric/Absorbance

Huelseweh et al. [109] developed an application for multiplex detection of biothreat agents based on the ArrayTube platform manufactured by Abbott (formerly Alere Technologies GmbH, Jena, Germany). In this setup, a microarray chip is attached to the bottom of the reaction vial and capture antibodies are immobilized to the glass surface of the microarray chip by means of an epoxy layer. Toxins present in the sample bind to the corresponding antibody spots during incubation time. All spots with bound analytes are detected by adding biotin-labeled anti-toxin antibodies and subsequently streptavidin-poly-horseradish peroxidase (SA-Poly-HRP) conjugate, which is made visible by adding HRP-substrate. A 5-plex assay is established including the security-sensitive toxins ricin and SEB. Within a total analysis time of 60–90 min, LODs of 0.1 ng/mL and 0.2 ng/mL were achieved for ricin or SEB, respectively. The platform applied here is potentially suited to mobile laboratory settings; however, many manual manipulation steps are still mandatory in this assay protocol. Therefore, an on-site application by minimally trained personnel is not feasible for this technology. In the following years, the ArrayTube platform was applied to many clinical and veterinary diagnostics issues; however, further applications in the biodefense market were not described.

Bertin Technologies (Montigny-le-Bretonneux, France) developed a platform called KIM based on magnetic field enhanced immuno-agglutination technology [110,111]. Capture as well as detection antibodies are covalently coupled to magnetic beads (200 nm diameter). Due to frequent turning on and off (mixing) of a magnetic field, the likelihood of sandwich formation is increased. Finally, aggregation of magnetic beads is measured as optical density with a laser. Thereby, the magnitude of magnetic beads aggregation is proportional to the target concentration. The device was integrated in a small case, ruggedized and is therefore optimally suited as a field unit for on-site confirmation of the presence of security sensitive toxins in the hotzone. Assays for security sensitive toxins BoNTs, SEB and ricin have been in development and achieved good sensitivities in the low to mid ng/mL range within approx. ten min assay time [57]. However, the device was designed only for single-plex measurement and the device is not commercially available any more.

##### Fluorescence

Jenko et al. [112] developed an ELISA-based protein antibody microarray to detect the ten proteotoxins BoNT serotypes /A to /F, ricin, shiga toxins 1 and 2 as well as SEB simultaneously. The screening and use of optimal antibody pairs together with the biotin–tyramide signal amplification system results in assays capable of detecting proteotoxins down to pg/mL levels in clinical and environmental samples [112]. However, the established assays are not automated, i.e., manual washes and reagent additions have to be performed as well as fluorescent readout being done with a laboratory-based commercial system. Furthermore, the total analysis time is at least 120 min. Therefore, the developed method is not suited for on-site detection purposes; however, it is a valuable tool to provide fast multiplexed screening and confirmatory identification after a potential bioweapon incident in a laboratory environment with trained personnel.

Peruski et al. [113] already developed in 2003 an approach for detection of the security sensitive toxins BoNT/A and /B as well as SEB (and *Francisella tularensis*) based on the commercialized dissociation-enhanced lanthanide fluorescence immunoassay-time-resolved fluorometry (DELFIA^®^-TRF) assay system (Perkin-Elmer Life Sciences, Akron, OH, USA). Biotin-labeled antibodies were immobilized to streptavidin-coated 96-well microtiter plates. After binding of the analyte, a lanthanide (Eu^3+^)-labeled detection antibody is added producing a signal after the addition of an enhancement solution. During this step, lanthanide label dissociates from the antibodies and free labels form stable chelates of high fluorescence intensity. Within approx. 2 h assay time, LODs between 4 and 20 pg/mL were achieved. The authors showed also the identification of proteotoxins in complex clinical (serum, urine) as well as environmental matrices (dirt, sewage). One advantage of DELFIA^®^-TRF is that background fluorescence of the sample does not influence the measurement. However, due to the use of a conventional microtiter plates assay with quite a few manual processing steps, the requirement of a benchtop microplate fluorescence reader as well as an assay time of 2 h, the developed assay is up to now not suitable for on-site multiplex detection.

Weingart et al. [114] developed a new microfluidic platform named the Inca Bioanalytical System for the simultaneous detection of BoNT/A, SEB and ricin. The platform is based on a so-called IncaSlide, a microchanneled slide exhibiting capture antibodies immobilized photochemically, which is connected to a fluid propagation system as well as a reservoir. The sample containing the target molecules is pumped through the microchannels and corresponding targets bind to the immobilized capture antibodies. The sandwich is formed by the addition of biotin-labeled detection antibodies and a fluorescence signal is generated by the subsequent addition of Cy5-dye conjugated to streptavidin. Fluorescence is read by scanning the slides. The developed platform enabled the detection of 0.5 ng/mL of BoNT/A, ricin, or SEB in buffer, whereas LOD for BoNT/A and ricin in raw milk is 1 or 5 ng/mL, respectively. The total analysis time of the system is approx. 90 min.

**Figure 2:**
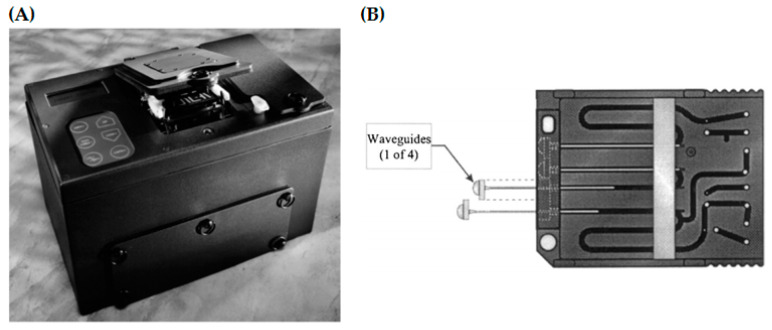
(**A**) Picture of the portable, four channel fluorimetric RAPTOR^TM^ assay system (Reprinted from Biosensors and Bioelectronics, 14, Anderson, G.P.; King, K.D.; Gaffney, K.L.; Johnson, L.H., Multi-analyte interrogation using the fiber optic biosensor, 771–777, Copyright (2000), with permission from Elsevier). (**B**) Scheme of a RAPTOR^TM^ assay coupon depicting orientation of optical fibers (Reprinted from Biosensors and Bioelectronics, 14, Anderson, G.P.; King, K.D.; Gaffney, K.L.; Johnson, L.H., Multi-analyte interrogation using the fiber optic biosensor, 771–777, Copyright (2000), with permission from Elsevier).

In contrast to the before mentioned techniques, the multiplex platforms NRL Array Biosensor [115,116], the fiber optic fluorometer RAPTOR^TM^ [56,117] and the QTL Biosensor^TM^ 2200 R [56] are clearly designed for rapid and simultaneous on-site detection of several proteotoxins, namely BoNT/A, BoNT/B, ricin and SEB. In the meantime, the QTL Biosensor^TM^ 2200 R was already discontinued. The portable QTL Biosensor^TM^ 2200 R (developed by QTL Biodetection, LLC, Santa Fe, NM, USA; distributed in USA by MSA, Cranberry Township, PA, USA) used fluorescence emission as detection principle for identification of proteotoxins. Capture antibodies are conjugated to magnetic beads carrying a fluorescent polymer. Binding of the target analyte to the capture antibodies releases quencher molecules bound to the fluorescent polymer and, thus, causes a fluorescence increase in the presence of proteotoxins. Total assay time is approx. 10 min, achieving LODs in the low to mid ng/mL range for the proteotoxins BoNT/A, ricin and SEB [56]. The multiplexing capability of this system is limited to four analytes. Furthermore, the system can be susceptible to matrix interferences due to the fact that the surface chemistry cannot completely avoid non-specific binding [56]. The basics of the RAPTOR^TM^ biosensor (Figure 2A) commercialized by Research International (Monroe, WA, USA; https://www.resrchintl.com/) were developed by Ligler and co-workers [118,119]. Antibodies are immobilized on optical fibers and, in the presence of the corresponding analyte, fluorescently labeled detection antibodies bind in a sandwich assay format. The RAPTOR^TM^ system has four channels with specially tapered optical fibers (Figure 2B), therefore enabling the detection of up to four analytes simultaneously in an automated procedure within 3–10 min. For ricin and SEB, LODs of 50 ng/mL or 10 ng/mL were determined in a 4-plex assay (two further targets in this study were bacterial agents) [91].

Another research approach of the Ligler group at the Naval Research Laboratory led to the NRL Array Biosensor based on planar waveguide arrays. Capture antibodies are immobilized on glass slides which are modified with a silane-based self-assembled monolayer. After binding of the analyte and subsequent binding with detection antibodies conjugated with fluorescent labels, presence of analyte is detected using evanescent wave excitation monitored with a CCD camera. The system allows the simultaneous detection of up to nine analytes within 15 min assay time achieving LODs of 8 ng/mL for ricin, 0.1 ng/mL for SEB, 20 ng/mL for BoNT/A toxoid, 200 ng/mL for BoNT/B toxoid and 1.6 ng/mL for cholera toxin [115]. Furthermore, the NRL Array Biosensor allows parallel detection of BWA-relevant bacteria, viruses and toxins with one platform applying one microarray [115]. A microfluidic electrophoretic chip-based immunoassay was developed for parallel detection of ricin, Shiga toxin 1 and SEB within less than 20 min [120]. A portable, self-contained device was created with the microfluidic chip integrated together with miniaturized electronics, optical elements, fluid-handling components and data acquisition software. Detection is realized in a sandwich immunoassay format applying fluorescently labeled detection antibodies and laser-induced fluorescence detection. Furthermore, electrophoretic separation of antibody–analyte complex and excess antibody is performed with polymeric gels integrated in the microfluidic chip. The method achieved LODs of 1.32 µg/mL, 8.4 ng/mL and 35 ng/mL for ricin, SEB or shiga toxin 1, respectively. Reliable high volume manufacturing and stability of in situ gels for electrophoretic separation is a problem due to quite frequent bubble formation in the gel during photopolymerization. In contrast to highly sensitive lab-based techniques such as multiplex ELISA [112] or DELFIA-TRF [113] assay, these systems are less sensitive, with LODs in the low to medium ng/mL range; however, they are portable and operable by untrained users. Furthermore, these assays exhibit a more rapid assay time (15 min compared to 2 h) which is a prerequisite for on-site application by first responders wearing full protective equipment.

**Figure 3:**
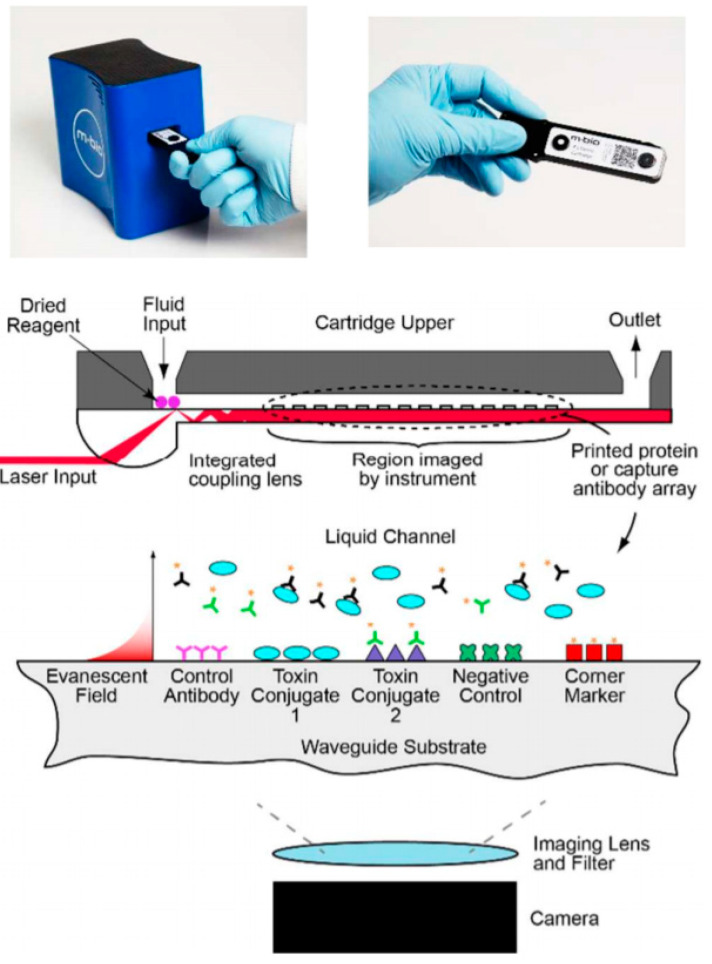
Portable MBio MQ reader and disposable cartridge (**Top**). Schematic representation of LightDeck^®^ technology (**Bottom**) (Reprinted with permission from [85]. Copyright (2018) American Chemical Society).

The LightDeck^®^ technology developed by MBio Diagnostics (Boulder, CO, USA; https://mbiodx.com/) relies on planar waveguide microarray technology and fluorescence imaging. Illumination is done by a solid-state diode laser (639 nm). A disposable injection-molded plastic cartridge contains a laser light-coupling lens which directs the laser light down the plastic substrate. The multimode waveguide generates an evanescent illumination field at the microarray surface. Due to the evanescent field illumination, interference from cells or unbound fluorophores are minimized allowing one to conduct assays in the presence of complex sample matrices without washing steps [85,86]. Bickman et al. [85] demonstrated the application of this planar waveguide technology for duplex detection of low molecular weight toxins microcystin and cylindrospermopsin applying a fluorescent competitive immunoassay in a disposable cartridge. The disposable cartridge is analyzed using the portable and battery powered MBio MQ reader (Figure 3). An automated assay workflow requiring 10 min assay time achieved LODs of 0.4 ng/mL for microcystin and 0.7 ng/mL for cylindrospermopsin [85]. Up to now, the technology was applied only for environmental detection purposes; however, it exhibits potential also for multiplex detection of security sensitive proteotoxins and low molecular weight toxins in the field of biosecurity.

Soares et al. [121] developed a new portable fluorescent immunoassay to detect the low molecular weight toxins aflatoxin B1, ochratoxin A and deoxynivalenol. For the multiplex, bead-based microfluidic competitive immunosensor Si:H thin film photodiodes are applied to detect fluorescence signals. This novel platform achieved LODs in the range of 1 ng/mL for all tested mycotoxins in a single-step assay and within 1 min after sample addition [121]. Robustness towards sample matrices as well as versatility to detect proteotoxins have to be demonstrated in the future.

##### Cytometry/Bead-Based Assays

Recent advancements using the xMAP^®^ technology developed by Luminex Corp. (Austin, TX, USA; https://www.luminexcorp.com/) offer a ELISA system based on magnetic fluorescent beads as solid support with multiplex capability [122]. This technique applies color-coded (magnetic) microspheres with capture antibodies coupled to these beads as solid support for the ELISA, whereas the detection antibody is labeled via biotin–streptavidin interaction with the fluorescent reporter phycoerythrin. Readout of fluorescence from beads as well as a reporter excited by light-emitting diodes is done by a charged coupled device (CCD) camera and facilitates the measurement of the median fluorescence intensity (MFI) of each sample. Pauly et al. [95] established a multiplexed magnetic suspension bead array for simultaneous detection of BoNT/A and /B, ricin, abrin as well as SEB. The authors combined a novel immunization strategy to generate high-affinity monoclonal and polyclonal antibodies against the proteotoxins together with Luminex xMAP^®^ technology. The obtained LODs (2 pg/mL for ricin, 3 pg/mL for SEB, 21 pg/mL for BoNT/A, 73 pg/mL for BoNT/B as well as 546 pg/mL for abrin within 210 min assay time) demonstrate the feasibility to detect biothreat agents toxins from a 50 mL sample with a previous 2 h enrichment incubation step utilizing the magnetic property of antibody labeled fluorescent beads. The aim of the study was the development and validation of a lab-based platform for multiplex and highly sensitive detection of the proteotoxins. Therefore, it is obvious that this multiplex approach requires several manual steps, is not portable and is thus not suited for an on-site detection approach [95]. Recently, the same group established on the Luminex xMAP^®^ platform an assay to detect catalytically active BoNTs. Therefore, the authors developed a panel of monoclonal neoepitope antibodies specific for the newly generated N- and/or C-termini of the substrate cleavage products of BoNT serotypes A to F. These antibodies were applied in three duplex assays to discriminate BoNT/A to /F achieving LODs in the range of 0.3–80 pg/mL with a total assay time of approx. 21 h [123]. Particularly, the catalytic cleavage reaction required an incubation time of 18 h to achieve LODs in the pg/mL range. A more rapid multiplex assay (approx. 50 min assay time) for the detection of proteotoxins abrin, BoNTs, ricin, SEA, SEB and SEC based on xMAP^®^ technology was developed by Garber et al. [83]. Due to a significantly shorter incubation time (<1 h vs. 3.5 h or 16 h with enrichment) and reduced sample volume, sensitivity was reduced compared to the assay developed by Pauly et al. [95]. LODs in the range of mid ng/mL to high ng/mL for mentioned toxins in food samples, such as chocolate milk, infant formula or vegetable juice, were achieved. Relying on these still acceptable LODs compared to human LD_50_ values of the analyzed toxins as well as a short assay time and high multiplexing degree, this approach shows potential for usage in mobile laboratory settings for the screening of suspicious food samples. Similarly, Simonova et el. [124] developed a suspension assay based on xMAP^®^ technology for the simultaneous detection of six proteotoxins (SEA and SEB, cholera toxin, ricin, BoNT/A and heat labile toxin of *Escherichia coli*). Within a total assay time of approx. 3 h, the developed multiplex assay achieved LODs of 0.01 ng/mL for SEA, cholera toxin, BoNT/A and ricin as well as 0.1 ng/mL for SEB and heat labile toxin in buffer (1% BSA in PBS). LODs for the mentioned toxins in milk were 2–5 fold increased, demonstrating the robustness of the developed assay. Based on this work, the authors developed additionally a screening assay for the detection of SEA, SEB and toxic shock syndrome toxin (TSST) in *Staphylococcus aureus* culture supernatants [125]. As for the other assays developed based on xMAP^®^ technology, manual wash steps and reagent additions have to be executed by the user. Furthermore, the analysis time of approx. 3 h will conflict with an on-site use of this kind of technique. However, on the one hand, there is potential for shortening the assay time, as shown by Garber et al. [83]; while on the other hand, it is well suited for application in confirmatory laboratories or even mobile laboratory settings. Furthermore, xMAP^®^ technology was already integrated in an environmental monitoring system as identification platform for security sensitive pathogens [126]. This was a first proof that xMAP^®^ technology can be used in autonomous identification platforms in mobile settings. The company Luminex offers also a commercially available multiplex assay to rapidly detect and identify six biothreat toxins (BoNT/A, BoNT/B, BoNT/E, BoNT/F, ricin and SEB) called xMAP Biothreat Toxin Panel.

**Figure 4:**
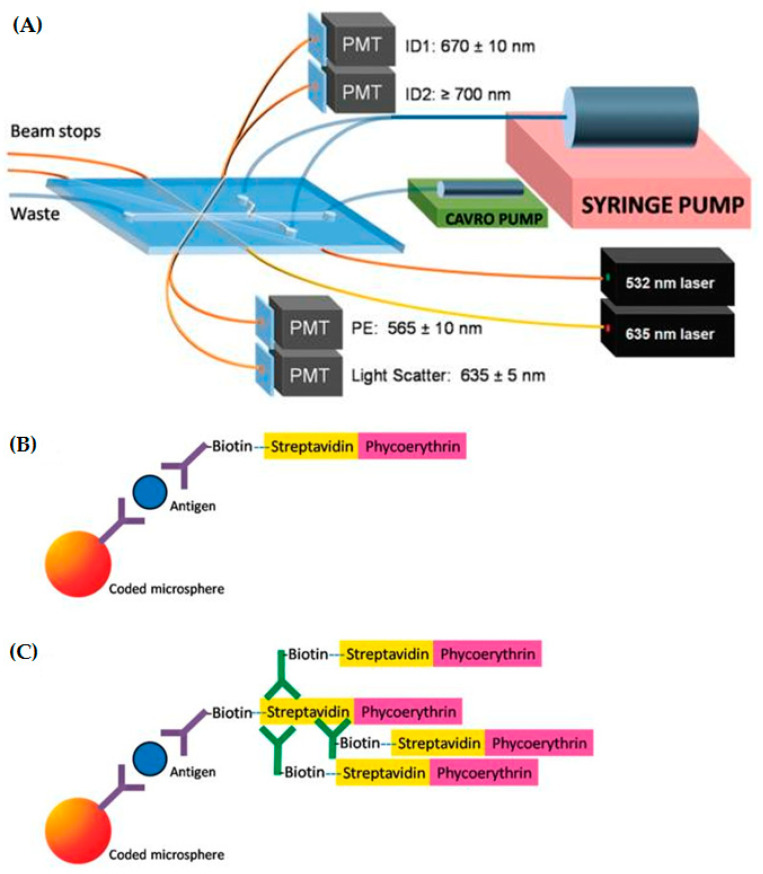
(**A**) Schematic layout of the microcytometer for multiplex detection of security sensitive toxins. The syringe pump provides sheath flow, while the CAVRO^®^ syringe pump was used to inject samples into the microfabricated channel of the polydimethylsiloxane (PDMS) chip. Depicted cables into the PDMS chip guided 635 and 532 nm laser light into the interrogation region and guided excess light out of the beam stops. Additional fiber optics directed emission light to four separate photo-multiplier tubes (PMTs) to collect microsphere ID fluorescences (670 ± 10 nm and ≥700 nm), light scatter (635 ± 5 nm) and phycoerythrin fluorescence (565 ± 10 nm). Sizes are not to scale (Reprinted with permission from [127]. Copyright (2009) American Chemical Society). (**B**) Schematic representation of conventional sandwich immunoassay performed on microspheres (Reprinted with permission from [127]. Copyright (2009) American Chemical Society). (**C**) Signal amplification approach applying additional biotin-labeled anti-streptavidin antibodies for enhanced streptavidin–phycoerythrin binding (Reprinted with permission from [127]. Copyright (2009) American Chemical Society).

Based on the xMAP^®^ technology, Ligler and co-workers developed a microcytometer employing fluorophore-labeled microspheres coupled to capture antibodies enabling the multiplex detection of security sensitive toxins (as well as BWA-relevant bacteria and viruses) in a sandwich immunoassay format (Figure 4). In contrast, to the bench-top devices from Luminex Corp. (MAGPIX^®^ or Luminex^®^ 200^TM^) the aim of the development was to provide a cost effective as well as really portable microcytometer. The prototype achieved LODs comparable to the commercial available bench-top device (1.6 ng/mL for cholera toxin, 0.064 ng/mL for SEB as well as 1.6 ng/mL for ricin) [127]. The applicability of such a microcytometer to detection of bacteria and toxins in a 10-plex sandwich immunoassay format in more complex sample matrices like serum or nasal swabs was later demonstrated successfully [128]. Despite these promising results, a commercialization of a portable microcytometer for multiplex immunoassay analysis is still pending.

##### Chemiluminescence/Electrochemiluminescence

One of the first commercial portable optical biosensor systems for the detection of security sensitive toxins, the BioVeris M1M system (BioVeris Corporation, Gaithersburg, MD, USA) [129] applies electrochemiluminescent labels once a sandwich composed of capture antibody, analyte and detection antibody is formed on a magnetic bead. The beads were brought over an electrode where a potential was applied causing emission of light from the electrochemiluminescent label. The system achieved LODs in the mid pg/mL to low ng/mL range applying 30 min assay time [56]. However, the BioVeris system [129] is not available any more for application in the field of biodefense since the merging of the company with the Roche Corporation.

Similar to the BioVeris system, Meso Scale Discovery (Rockville, MD, USA; https://www.mesoscale.com/) offers an electrochemiluminescence (ECL) signal detection platform consisting of a reader device (Sector^®^ PR2 1800) and a 96-well (9, 16 and 25 spot multiplex plates) plate-based immunoassay system, which was intended for use in mobile laboratories. The design of an ECL multiplex assay specific to the proteotoxins BoNT/A and SEB (as well as *Bacillus anthracis* spores) achieved LODs of 40 pg/mL for BoNT/A and 10 pg/mL for SEB within an assay time of approx. 3 h and the necessity of performing manual plate washings and reagent additions [130]. Similar assays for detection of ricin as well as abrin in food stuff have been described with LODs in the high pg/mL range with assay times of approx. 90 min [88,89]. However, the availability of the system for biodefense applications is limited to the USA [57].

**Figure 5:**
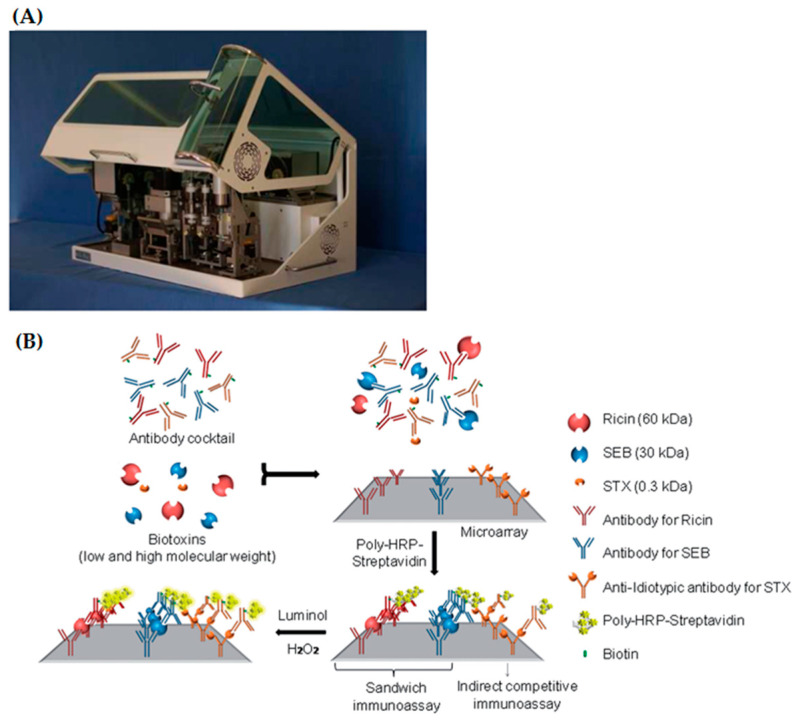
(**A**) Chemiluminescence instrument MCR3 for multiplex microarray analysis (Reprinted by permission from Springer Customer Service Centre GmbH: Springer, Analytical and Bioanalytical Chemistry [132], Copyright (2014)). (**B**) Schematic representation of sandwich (for SEB and ricin) and indirect competitive (for saxitoxin) immunoassays combined in the same microarray chip by using anti-idiotypic antibodies (Reprinted from [131]. Published by The Royal Society of Chemistry, 2014).

The prototype system Munich Chip Reader (MCR3) developed by the Technical University of Munich for applications in clinical diagnostics, food analysis as well as biothreat detection offers a microarray-based system with chemiluminescence detection showing potential for precise and fast on-site analysis. The principle of the system is a chemiluminescence sandwich immunoassay which is performed by using position specifically immobilized capture antibodies on microarray slides with corresponding biotin labeled detection antibodies. The signal generation occurs by means of the usage of SA-Poly-HRP complex as enzyme conjugate. The emitted chemiluminescence signal of luminol and peroxide is recorded by a CCD camera (Figure 5). Szkola et al. [131] applied this platform technology for simultaneous detection of the proteotoxins ricin and SEB as well as low molecular weight toxin saxitoxin (STX) in the field of biosecurity. Interestingly, the detection of the low molecular weight toxin saxitoxin was achieved by implementing monoclonal anti-idiotypic antibodies. Thus, a biosensor for simultaneous detection of proteotoxins and low molecular weight toxins was generated. The method achieved LODs of 2.9 ± 3.1 ng/mL for ricin, 0.1 ± 0.1 ng/mL for SEB and 2.3 ± 1.7 ng/mL for STX applying an optimized assay time of 18 min [131].

Furthermore, the authors applied the MCR3 system to the detection of solely low molecular weight analytes also with relevance in the field of biosecurity [133].

##### Digital Droplet Assays

Recently, digital droplet assays have generated enthusiasm for their ability to detect biomarkers with single-molecule sensitivity [134]. In digital assays, the signals from a single reporter molecule are compartmentalized in droplets and read independently. Because of the ability to detect single binding events, digital immunoassays generally have better sensitivity than their bulk counterparts. Up to now, these assays are mainly limited to laboratory settings due to the required instrumentation for generation, control and measurement of tens of millions of droplets/compartments. Yelleswarapu et al. [135] developed a optofluidic platform called microdroplet Megascale Detector (µMD) that miniaturizes digital assays into a mobile format. In a duplex digital ELISA capture, antibodies are coupled to color-coded beads and analytes are detected using a sandwich assay format applying HRP-labeled detection antibodies. Individual beads are encapsulated into droplets and readout if they have captured a single target protein. The mobile platform achieved with a duplex assay LODs of 0.0045 pg/mL and 0.0070 pg/mL for clinical relevant biomarkers GM-CSF and IL6, respectively [135]. Even if this platform was developed for clinical biomarker analysis, application of mobile digital droplet assays in the field of on-site detection of biothreat agents can have a great impact in future if the robustness of the method will be demonstrated.

#### 4.2.3. Cell-Based Biosensors

CANARY^®^ (Cellular Analysis and Notification of Antigen Risks and Yields) technology representing a cell-based biosensor was invented by scientists at the Massachusetts Institute of Technology Lincoln Labs and commercialized by PathSensors (Batimore, MD, USA; https://pathsensors.com/). The technique employs engineered B lymphocytes expressing the calcium-dependent bioluminescent protein aequorin. Furthermore, the B lymphocytes carry a membrane bound antibody for specific detection of a particular biothreat agent. Binding of an antigen to the membrane-bound antibody results in activation of an intracellular calcium ion channel and, thus, emitting of light by aequorin [136]. Light emission from engineered B cells sitting in spatially separated individual cuvettes is detected by a PMT. PathSensors developed several platforms, whereas the BioFlash^®^ Biological Identifinder for analyzing biothreat agents in air as well as the Zephyr for screening liquid and powder samples for the presence of biothreat agents are of particular importance in the field of biosecurity. In the most common setup, up to six biothreat agents (theoretically, up to 21 agents can be analyzed with one cartridge disc in parallel) can be detected simultaneously using the portable stand-alone BioFlash^®^ Biological Identifinder including the proteotoxins BoNT/A, ricin and abrin [90]. According to a validation study, the CANARY technology revealed an LOD of 3 ng/mL for ricin using 200 µL sample volume per test within a total analysis time of approx. 10 min [65]. However, a potential drawback of this technology is the limited stability of engineered B cells of only a few weeks.

#### 4.2.4. Electrochemical Biosensors

Because of the combination of highly specific antigen–antibody interaction as well as the high sensitivity of electrochemical methods, electrochemical immunosensors have achieved significant progress in various applications, among them the field of public security. Electrochemical immunosensors exhibit many advantages compared to other transduction technologies, including sensitivity, low-cost instrumentation, feasibility of miniaturization, potential for automation as well as operational simplicity [137].

**Figure 6:**
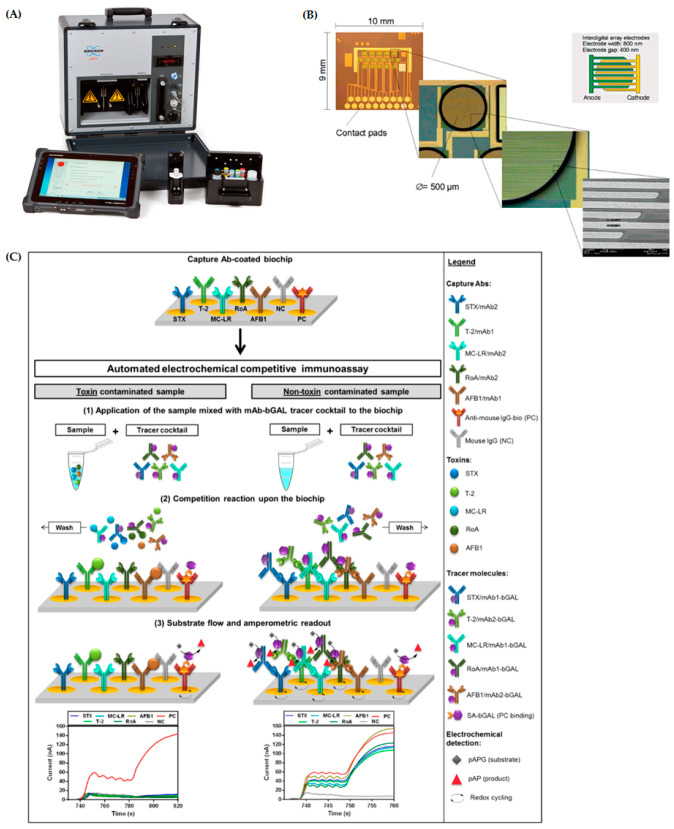
(**A**) Portable BioDetector integrated (pBDi) with sample holder and reagents holder as well a Tablet PC with control software running. (**B**) Electrochemical biochip layout with interdigitated electrode structure (Reprinted with permission from [141]. Copyright (2006) American Chemical Society). (**C**) Assay scheme of multiplex competitive immunoassay for detection of security relevant low molecular weight toxins (Reprinted from [94]. MDPI (2019)).

The pBDi system (portable BioDetector integrated; Bruker Optik GmbH, Ettlingen, Germany; https://www.bruker.com/) is an example for an electrochemical biochip-based detection platform for biothreat agent detection (Figure 6A). The first work on this microelectrode array platform was done at the Fraunhofer Institute for Silicon Technology (Itzehoe, Germany) [138]. In collaboration with Siemens AG (Erlangen, Germany) and Infineon Technologies AG (Munich, Germany), a 128 position sensor array chip [139] and a 16 position chip were developed [140,141] carrying interdigitated microelectrodes (Figure 6B). Quite early during development, the first applications based on this electrochemical biochip technology focused on multiplex detection of security sensitive toxins were developed using a prototype reader platform (ePaTox system) [142,143]. Based on these promising results, Bruker developed a portable and ruggedized platform for on-site usage called pBDi as well as several commercially available detection kits for the detection of security sensitive proteotoxins, bacteria and orthopox viruses [92,93]. The pBDi employs electrochemical biochip technology for multiplex fully automated ELISA-based detection of five to six agents simultaneously (for commercially available pBDi Test Kits; for customer specific assays in development, 12-plex immunoassays were already demonstrated). Capture antibodies are immobilized by piezo-based non-contact spotting on gold electrodes and facilitate the specific binding of corresponding analytes. Detection of bound analytes is realized by application of a biotin labeled detector antibody enabling binding of streptavidin-β-galactosidase conjugate. The reporter enzyme β-galactosidase converts an electro inactive substrate (p-aminophenyl-β-D-galactopyranoside; pAPG) into an electro active product (p-aminophenol; pAP) resulting in a detectable electrical signal. Oxidation of pAP to quinonimine provides the electrical signal. Redox cycling between interdigitated electrode design regenerates pAP. This redox cycling process leads to permanent increase of measured current [144]. Unique biochip cartridge design and small reaction volume on electrochemical biochips favor rapid reaction kinetics allowing biothreat agent identification in approx. 20 min (for proteotoxin detection). Sensitivity in the low ng/mL range for proteotoxin detection is achieved due to the high turnover of enzymatic reaction, and, in addition, the redox cycling procedure between pAP and quinoneimine due to interdigitated electrode structure built into the experimental procedure significantly contributes to the signal amplification.

The successful application of electrochemical biochip technology to 5-plex detection of BoNT/A, BoNT/B, BoNT/F, ricin and SEB was recently demonstrated in the European proficiency test EQuATox by the detection of toxins ricin or SEB in unknown samples [145,146]. Results demonstrated that electrochemical biochip technology (in this proficiency tests, the progenitor of the pBDi, the so called pTD—portable Toxin Detector—platform from Bruker Daltonik, Bremen, Germany was used) is able to detect qualitatively down to 0.5 ng/mL ricin in 0.1% (*w/v*) BSA/PBS buffer [145] as well as down to 0.5 ng/mL SEB in phosphate buffer containing BSA [146], whereas several LFIAs failed to detect these low concentrations of proteotoxins. Furthermore, the robustness of the developed electrochemical 5-plex assay was demonstrated by detection of proteotoxins in sample matrices such as milk, organic fertilizer or meat extract [145,146].

Recently, the detection capability of the system was broadened to security sensitive low molecular weight toxins [94]. Therefore, a five-plex assay for simultaneous detection of bioterrorism relevant low molecular weight toxins saxitoxin, microcystin-LR, T-2 toxin, roridin A and aflatoxin B1 was established on the pBDi platform. The developed assay relies solely on anti-idiotypic antibodies as epitope-mimicking reagents (Figure 6C). Thus, no potentially harmful toxin—protein conjugates usually mandatory for competitive immunoassays are required. LODs were 1.2 ng/mL, 1.5 ng/mL, 0.4 ng/mL, 0.5 ng/mL and 0.6 ng/mL for saxitoxin, microcystin-LR, T-2 toxin, roridin A or aflatoxin B1, respectively, applying a fully automated detection regime with a total assay time of 13.4 min. The robustness of the assay was demonstrated by analyzing human serum samples for the presence of the analyzed low molecular weight toxins without elaborate sample preparation steps [94].

Using a similar setup, Dill et al. [147] showed that CombiMatrix’s VLSI arrays consisting of individually addressable electrodes are applicable for multiplex biothreat agent detection (ricin as well as bacteria and other proteins). Applying a sandwich immunoassay format with HRP as reporter enzyme, an LOD of 0.3 ng/mL for ricin was achieved within an assay time of 12 min. However, the CombiMatrix platform in combination with the application to detect on-site biothreat agents was not commercialized.

In 2002, Kreuzer et al. [148] described an amperometric biosensor for the detection of several seafood toxins (such as okadaic acid, brevetoxin, domoic acid and tetrodotoxin). The biosensor employed disposable screen-printed carbon electrodes as well as alkaline phosphatase as reporter enzyme generating p-aminophenol, which is detected amperometrically. The authors demonstrate the capability of this biosensor to be used in the field for seafood toxin detection and achieved LODs in the low ng/mL range within 30 min total assay time.

#### 4.2.5. Others

**Figure 7:**
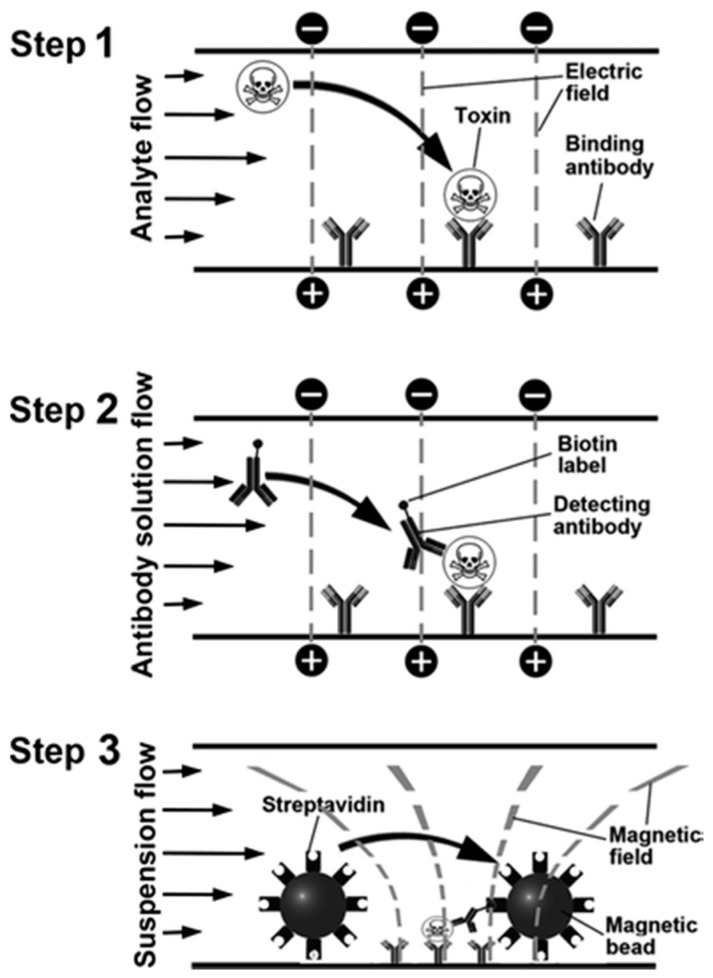
Schematic assay workflow for an “active” sandwich immunoassay relying on magnetic bead labels. Step 1: electrophoretic capturing of bacterial proteotoxins on a microarray solid surface from flow-through. Step 2: active electrophoretic labeling of the captured analyte by addition of corresponding biotinylated detection antibodies. Step 3: Microscopic detection of the microarray-bound biotin labels by addition of streptavidin-coated magnetic beads in a shear-flow and magnetic field (Reprinted with permission from [149]. Copyright (2012), American Chemical Society).

Magnetic particles can be used as labels that bind in a biologically specific manner to a surface and thereby report the presence of a specific molecular species. Shlyapnikov et al. [149] combined flows over a microarray surface exhibiting immobilized capture antibodies with electrophoresis and magnetophoresis to bind corresponding bacterial toxins to the microarray surface. Subsequently, magnetic bead labels were detected via microscopic inspection (Figure 7). Applying this assay principle, the detection of five bacterial proteotoxins (cholera toxin, heat-labile toxin of *Escherichia coli*, SEA, SEB and TSST from *Staphylococcus aureus*) in different sample matrices (i.e., buffer, water, milk and meat extract) was feasible within an approx. 10 min assay time. LODs for tap water as sample matrix were in the range of 0.1 to 1 pg/mL for the five different toxins. On the contrary, an LOD of 0.1 ng/mL was achieved without electrophoretic concentration of the toxins, demonstrating the importance of a forced transport of proteins at the recognition steps of the assay.

Based on results achieved with enzyme-based colorimetric immunofiltration columns (ABICAP^®^, Senova Gesellschaft für Biowissenschaft und Technik mbH, Weimar, Germany), Achtsnicht et al. [150] established a magnetic sandwich immunoassay inside a 3D immunofiltration column. Therefore, monoclonal capture antibodies specific for cholera toxin B subunit were immobilized on polyethylene matrix of the filtration column, whereas monoclonal detection antibodies were conjugated to superparamagnetic beads. The beads act as labels for the magnetic frequency mixing detection technique. An LOD of 0.2 ng/mL cholera toxin B subunit was achieved within approx. 2 h assay time. The method has the potential to be used on site; however, the degree of automation and assay time have to be improved. Furthermore, the multiplexing capability has to be demonstrated, even if there are promising initial results [151].

## 5. Conclusions

The use of highly sensitive and specific immunoassays for the laboratory-based detection of security sensitive toxins is a valuable and helpful decision-making tool in the case of a potential bioterroristic attack to identify the causing agent. Therefore, antibodies as biorecognition elements play an outstanding role even nowadays.

Several commercial products for multiplex detection of biological toxins in the field are available on the market. Most of them are based on LFIAs; others are based on protein microarray platforms with optical or electrochemical readout techniques. All of them are valuable tools for a rapid and profound assessment of a potential bioterroistic situation. However, all platforms have potential in regard to increasing multiplexing capacity, to broaden the detection capability to proteotoxins and low molecular weight toxins as well as the simplicity and robustness of the assay device and assay kit. Recent progress in antibody engineering and the usage of antibody fragments into immunoassays will further improve the stability and performance of these biosensors. Furthermore, nanomaterials and nanozymes show potential in improving target recognition and signal amplification. The development and optimization of fully automated microfluidic platforms will reduce operator errors due to the integration of sophisticated tasks, such as sample preparation, incubation steps, signal generation and detection. Furthermore, there is a great research effort toward multi modular technologies, i.e., platform technologies enabling one to run several assay types (immunoassay, nucleic acid testing) on one technological platform, broadening the application settings of these technological platforms. Applying these recent advances for the development of next generation commercial immunosensors will result in more rapid, robust and easy to operate sensor platforms exhibiting improved analytical and operational characteristics.

## Figures and Tables

**Table 1:** Comparison of toxicity (median lethal dose LD_50_ for laboratory mice) of security sensitive proteotoxins as well as low molecular weight toxins (adapted from [1,2]).

Toxin	Toxicity (LD_50_) [µg/kg]	Source	Chemical Structure ^1^	Classification
Botulinum neurotoxins (BoNTs)	0.001	Bacterium	Proteotoxin	Category A CDC ^2^ [3]; AG ^3^ [4]
Shiga toxin	0.002	Bacterium	Proteotoxin	AG [4]
Tetanus toxin	0.002	Bacterium	Proteotoxin	
Staphylococcal enterotoxin B (SEB)	0.02 ^4^	Bacterium	Proteotoxin	Category B CDC [3]; AG [4]
Diphtheria toxin	0.1	Bacterium	Proteotoxin	
Maitotoxin	0.1	Marine dinoflagellate	LMW	
Palytoxin	0.15	*Palythoa* corals and *Ostreopsis* dinoflagellates	LMW	
Ciguatoxin	0.25	Marine dinoflagellate	LMW	
Abrin	0.7	Plant	Proteotoxin	AG [4]
Textilotoxin	0.6	Snake venom	LMW	
*Clostridium perfingens* toxins	0.1–5.0	Bacterium	Proteotoxin	Category B CDC [3]; AG [4]
Batrachotoxin	2.0	Poison arrow frog	LMW	
Ricin	3.0	Plant	Proteotoxin	Category B CDC [3]; AG [4]; OPCW Schedule 1 ^5^ [5]
α-Conotoxin	5.0	Cone snails	LMW	AG [4]
Taipotoxin	5.0	Snake	LMW	
Tetrodotoxin	8.0	Pufferfish	LMW	AG [4]
α-Tityustoxin	9.0	Scorpions	LMW	
Saxitoxin	10.0	Marine dinoflagellate	LMW	AG [4]; OPCW Schedule 1 [5]
Staphylococcal enterotoxin B (SEB)	10.0 ^6^	Bacterium	Proteotoxin	Category B CDC [3]; AG [4]
Anatoxin-A	50.0	Blue-green algae	LMW	
Microcystins	50.0	Blue-green algae	LMW	AG [4]
Aconitine	100.0	Plant	LMW	
T-2 toxin	1.210.0	Fungus	LMW	AG [4]
		For comparison, synthetic substances		
VX	15.0	Nerve agent		
Soman	64.0	Nerve agent		
Sarin	100.0	Nerve agent		

^1^ Proteotoxin or LMW (low molecular weight toxin), respectively. ^2^ Bioterrorism agents are classified by the Centers for Disease Control and Prevention (CDC, Atlanta, GA, USA) into three categories depending upon their ease of dissemination and the ability to cause excessive morbidity and mortality. Category A includes agents that have been used as a weapon of mass destruction exhibiting high morbidity and mortality (e.g., *Variola major* virus or *Yersinia pestis*). Category B agents are easy to disseminate and produce moderate morbidity and low mortality. Category C agents include emerging pathogens that could potentially be engineered for future mass dissemination. ^3^ Listed in the Australia Group (AG) List of human and animal pathogens and toxins for export control. ^4^ Predicted human aerosol. ^5^ Listed by the Organization for the Prohibition of Chemical Weapons (OPCW) as a controlled chemical under Schedule 1 compounds. ^6^ Aerosol nonhuman primates.

**Table 2:** Overview of key characteristics of portable platforms for multiplex detection of security sensitive toxins (adapted from [54,55]).

Feature	Minimal Requirement	Optimal Requirement
Scope of the Platform		
Intended use case	Multiplex identification of panels of security sensitive toxins	Multiplex identification of proteotoxins and low molecular weight toxins, plus identification of biological warfare agent (BWA)-relevant bacteria, spores and viruses
Operation site	Mobile laboratory (functioning laboratory with trained personnel, inconsistent electricity supply, limited climate control)	On-site, i.e., in the hot zone (minimally trained staff, no electricity, no climate control, dust)
User	Trained personnel, i.e., specialized personnel for BWA detection in fire brigades, analytical task forces or military	Minimally trained personnel, i.e., first responders
**Instrument**		
Instrument design	Single integrated instrument with port(s) for reading one or more multiplex assays (cartridges) for simultaneous detection of multiple security sensitive toxins	
Size	Small, portable instrument (approx. 50 cm × 50 cm × 25 cm or smaller)	
Weight	≤15 kg	≤4.5 kg
Power requirements	Local 110–220 AC mains power, plus uninterruptable power supply plus rechargeable battery with 4-h operation	Same, with rechargeable battery (8 h operation)
Throughput	Up to 8 sample runs per instrument per 8 h day	Up to 40 sample runs per instrument per 8 h day
Environmental stability-Operating range of platform	Operation at 10–35 °C and up to 90% non-condensing humidity	Operation at 5–45 °C and up to 90% non-condensing humidity
Biosafety	Closed, self-contained system; easy decontamination of instrument surface as well as possibility for decontamination of whole instrument using formaldehyde or hydrogen peroxide fumigation	
Training	<2 days training for minimally skilled staff	<1 day training for minimally skilled staff
Operation	<1% operation error for a trained user Operation in full protective equipment Automated analysis and interpretation of measurement data	
Calibration	Need for instrument calibration on-site on a yearly basis by minimally trained technician	Self-check alerts operator to instrument errors or warnings; no calibration needed
Result readout	Qualitative result available to user sufficient to inform responsible person for decision-making	Same, plus quantitative result for each analyte
Data display	On-instrument or on a separate reading device (mobile phone, tablet PC) with ability to function in various light conditions. Generation of report file with information about sample ID, operator ID, date, location, assay applied etc.	
Connectivity	USB, integrated Local Area Network (LAN) port, integrated Wi-Fi	Same, plus integrated Bluetooth, multi-band Global System for Mobile Communications (GSM) chipset 2G, 3G, LTE, 5G
Manufacturing	ISO 9001:2015 compliant	ISO 13485:2016 compliant
List price of instrument	≤$50,000 (USD)	≤$10,000 (USD)
**Assay Cartridge**		
Analytes	Simultaneous detection of proteotoxins as well as low molecular weight toxins from a single sample using one or more assay cartridges	Simultaneous detection of proteotoxins as well as low molecular weight toxins from a single sample using one universal cartridge; additional analyte detection capabilities preferred (e.g., nucleic acid testing for determination of presence of toxin producing organism)
Multiplexing capability	Analysis of one sample for the presence of six security relevant toxins at the same time in one or more assay cartridges	Analysis of one sample for the presence of 15 security relevant toxins at the same time in one or more assay cartridges
Test kit	All materials required for the assay, including assay cartridge, reagents and buffers included in individually packaged test kits	
Additional third-party consumables	None, except for sample collection and sample preparation	Cartridges contain all required reagents
Sample type	Ability to accept a wide range of environmental (e.g., soil, dust), food (e.g., milk, water), powder (e.g., bentonite, kaolin) as well as clinical (e.g., serum, whole blood, urine, nasopharyngeal swabs) samples	
Sample volume	The minimal sample volume required to reach relevant sensitivities (up to 1 mL acceptable)	
Sample preparation	Minimal sample preparation; no more than 3 steps such as pipetting, filtration or other off-cartridge-based steps acceptable	All sample preparation steps are integrated and performed within the assay cartridge; no precision steps required to be performed by the user
Limits of detection (LOD) in multiplex format	Achieving LODs in the range of LD_50_ of the security sensitive toxins	Equivalent (or improved) relative to reference assays
Specificity—inclusivity	Detection of all congeners of a toxin group or subtypes/ isoforms of a proteotoxin, respectively	
Specificity—exclusivity	No significant cross-reactivity with closely-related proteins or molecules outside the scope of security sensitive toxins	
Interfering substances	No interference for an individual analyte or mixtures of analytes because of interfering substances	
Time to result	<60 min	<20 min
Internal process controls	Internal process control must be integrated into the assay design	
Positive/Negative controls	External positive and negative controls are not required for each test but are performed on a regular basis	External positive and negative controls are not required for each test and do not need to be run on a regular basis
Environmental stability–transportation	No cold chain requirements; stable at 2–45 °C for up to 7 days, can tolerate short term temperature fluctuations from 0–50 °C	No cold chain requirements; stable at 2–45 °C for up to 15 days, can tolerate short term temperature fluctuations from 0–50 °C
Environmental stability–Operating range	10–35 °C	5–45 °C
Shelf life and storage conditions	12 months from date of manufacture at up to 25 °C	18 months from date of manufacture at up to 30 °C
Manufacturing	ISO 9001:2015 compliant	ISO 13485:2016 compliant
List price of assay per sample	≤$50 (USD)	≤$20 (USD)

**Table 3:** Overview of commercially available multiplex lateral flow (immunochromatographic) immunoassays (LFIAs) for detection of security sensitive toxins. Only the detectable toxin agents are noted, i.e., residual agents are BWA-relevant bacteria or viruses.

Name	Manufacturer	Detectable Toxin Agents	Multiplexing	Sample Volume [µL] ^1^	Read Out	Reader Available
Pro Strips^TM^	AdVnt (Phoenix, AZ, USA)	BoNTs (A&B) Ricin SEB	5	600	visual	Y
RAID^TM^ 5	Alexeter (Wheeling, IN, USA)	BoNTs Ricin SEB	5	400	visual	Y
RAID^TM^ 8	Alexeter (Wheeling, IN, USA)	BoNTs Ricin SEB	8	800	visual	Y
NIDS^®^ 4-Plex	ANP Technologies (Newark, DE, USA)	BoNT/A BoNT/B Ricin SEB	4	120	visual	Y
BioThreat Alert^®^	Tetracore (Rockville, MD, USA)	Abrin BoNTs Ricin SEB	4	150	visual	Y
IMASS^TM^ (discontinued)	BBI Detection (Crumlin, UK)	BoNTs Ricin SEB	8	2500	visual	N
Prime Alert^®^	Genprime (Spokane, WA, USA)	BoNTs Ricin SEB	3	250	visual	N

^1^ Volume requirement according to [65].

**Table 4:** Overview of commercially available multiplex microarray platforms for detection of security relevant toxins in the field exhibiting assay times < 60 min.

Name (Technology)	Multi-Plexing Capacity ^1^	Detect-Able Toxin Agents ^2^	LOD	Time [min]	Ready-to-Use Kits	Mobile Lab Use	On-Site Use ^3^	Ref
xMAP technology^®^, Luminex (Optical bead-based suspension array)	≤50 ^4^	Abrin, BoNTs, SEs, ricin, STX (as well as several further toxins) ^5^	pg/mL to low/ mid ng/mL ^6^	50	Y	Y	N	[83,84]
LightDeck^®^, MBio^®^ Diagnostics (Planar waveguide microarray)	≤60	MC, CYN, NOD; DA, STX; TTX,	Low ng/mL	~10	Y	Y	Y	[85,86,87]
Sector^®^ PR2 1800, Meso Scale Discovery (ECL)	≤25	Abrin, BoNTs, SEB, Shiga toxin, Ricin, T-2, STX	pg/mL to low ng/mL	15–90	Y	Y	N	[11,88,89]
CANARY^®^, PathSensors (Cell-based biosensor)	≤21	Abrin BoNT/A Ricin	Low ng/mL	2–10	Y	Y	Y	[65,90]
RAPTOR^TM^, Research Intl. (Optical fiber array)	4	Ricin, SEB	Mid ng/mL range	3–10	Y	Y	Y	[91]
pBDi, Bruker Optik GmbH (Electro-chemical biochip)	≤16	Abrin, BoNT/A, /B, /C, /D, /E, /F, ricin, SEA, SEB, AFB1, MC, RoA, STX, T-2	Low ng/mL	13–20	Y	Y	Y	[92,93,94]

^1^ The maximal or theoretical multiplexing capacity of the technology is noted. In general, the majority of referenced publications describe 3- to 6-plex immunoassays for detection of security sensitive toxins. ^2^ Abbreviations: BoNT: botulinum neurotoxin, SE: staphylococcal enterotoxin, STX: saxitoxin, MC: microcystins, CYN: cylindrospermopsin, NOD: nodularins, DA: domoic acid, TTX: tetrodotoxin, AFB1: aflatoxin B1, RoA: roridin A, T-2: T-2 toxin. ^3^ On-site detection capability means that the system is at least portable, i.e., a weight of ≤15 kg, size allows hand carrying, battery operation is feasible, operational robustness (ruggedization) is ensured as well as analysis time < 60 min. ^4^ Valid for the MAGPIX^®^ instrument. ^5^ Variety of customer-made applications due to open platform character of xMAP^®^ technology are described (see text for further literature). ^6^ Significant improvement of LODs down to low pg/mL range can be achieved by magnetic xMAP^®^ technology, as well as increased incubation times as described by [95].
